# Effect of Fortification with Mushroom Polysaccharide β-Glucan on the Quality of Ovine Soft Spreadable Cheese

**DOI:** 10.3390/foods11030417

**Published:** 2022-01-31

**Authors:** Efthymia Kondyli, Eleni C. Pappa, Dimitris Arapoglou, Maria Metafa, Christos Eliopoulos, Cleanthes Israilides

**Affiliations:** 1 Dairy Research Department, Institute of Technology of Agricultural Products, Hellenic Agricultural Organization-DEMETER, Katsikas, 45221 Ioannina, Greece; pappa.eleni@yahoo.gr; 2 Institute of Technology of Agricultural Products, Hellenic Agricultural Organization-DEMETER, 1 S. Venizelou, 14123 Lycovrysi, Greece; dimarap@yahoo.com (D.A.); mariametafa@gmail.com (M.M.); chris_eliopoulos@hotmail.com (C.E.); cisrailides@yahoo.gr (C.I.)

**Keywords:** fresh, soft, spreadable, cheese, β-glucan, mushroom

## Abstract

In the present work, a fresh spreadable cheese from ovine milk with or without (control) fortification with β-glucan was manufactured. β-Glucan was extracted from the mushroom *Pleurotus ostreatus* and its concentration in the cheese was 0.4% (*w*/*w*). The composition, biochemical, and sensory properties of the cheeses during 21 days of storage were determined. At the end of storage, cheese fortified with beta-glucan had 75.26% moisture content, 10.30% fat, 1.71% salt, and 8.50% protein. Generally, the addition of β-glucan at this concentration did not significantly affect the composition, color, and viscosity measurements or the level of proteolysis and lipolysis and the antioxidant activity of the cheeses. However, cheese fortified with β-glucan showed a higher moisture content than control cheese on the 1st and 21st day of storage while the levels of proteolysis and the sensory properties of the cheeses were unaffected. During the sensory evaluation, panelists evaluated cheese with β-glucan with higher scores regarding the flavor characteristic compared to control cheese. The major free fatty acid was acetic acid in both cheeses and its concentration was higher in cheese with β-glucan. The results of the present study could be used by the dairy industry for manufacturing new products with improved health benefits.

## 1. Introduction

Nowadays, consumers’ demand for healthy foods has encouraged the food industry to manufacture products with health benefits. Functional foods fulfill the basic human nutrients and have a potential positive impact on health when consumed in sufficient quantities. Their consumption is associated with health benefits regarding the prevention and control of chronic diseases. In the last years, there has been an increase in the demand for functional foods due to their beneficial effects [1,2].

β-Glucan is a naturally occurring polysaccharide of D-glucose monomers linked by β-glycosidic bonds. It is extracted either from cereals, mainly barley and oat, or from other sources, i.e., mushroom, algae, yeasts, bacteria, and seaweed. In cereals, β-glucan is a polymer of D-glucose with β (1→3) and β (1→4) linkages while, in non-cereal sources, this compound is found to have a linear chain of D-glucose with β (1→3) and β (1→6) linkages. β-Glucan is considered a functional compound and it is receiving increasing interest because it can promote health. The beneficiary effects of its consumption include reduced serum cholesterol, lower glycemic values, improved insulin resistance, improvement of gut health, and prevention of coronary heart and other diseases [3,4,5,6]. A level of 3 g/day has been approved by the Food and Drug Administration (FDA) regarding β-glucan’s decreased blood cholesterol effect [7]. The biotechnological addition of β-glucans to foods and animal feed and their functionality in dairy products was recently reviewed [8,9].

Medicinal mushrooms have been considered as functional foods only in recent years, although their use was a common and ancient practice, especially in Eastern countries, since long ago [10,11]. The β-glucans in medicinal mushrooms stimulate the immune system and show anticarcinogenic activity [12]. β-Glucans from mushrooms are used as supplements (tablets and capsules) or as feed for chicken and fish [8]. In the literature, the incorporation of β-glucan derived from different mushrooms (*Pleurotus ostreatus*, *Lentinula edodes*, *Pleurotus citrinopileatus*) in dairy products, such as yoghurt and cheese, has been reported in few publications [13,14,15].

Fresh cheeses can be consumed immediately after their manufacture. Some well-known types of fresh cheeses are Quark and cream cheese [16]. In Greece, popular soft spreadable cheeses are Galotyri and Katiki Domokou.

Therefore, the aim and the novelty of the present work was to study the production of a fresh, soft, and spreadable cheese from ovine milk fortified with β-glucan extracted from *Pleurotus ostreatus* mushroom and to determine its composition, biochemical characteristics, and sensory properties.

## 2. Materials and Methods

### 2.1. Extraction of Beta Glucan

The method of Wang and Zhang [17] with some modifications was used for the extraction of β-glucan from mushrooms of the species *Pleurotus ostreatus*. In detail, the mushroom was initially cut into pieces, lyophilized, and powdered. The lyophilized powder was treated with ethanol for 8 h using a Soxhlet extractor. The residue was soaked in 0.9% NaCl solution (at 70 °C for 24 h) and was centrifuged (at 5700 rpm for 10 min). The resultant residue was extracted with 1 M NaOH (at 40 °C for 8 h). The supernatant was then neutralized with 1 M acetic acid. The precipitate, which was β-glucan, was collected and washed with distilled water several times to obtain a white color. β-Glucan in this stage is referred to as β-glucan in the form of a paste.

β-Glucan in the form of a paste was first dispersed in a small quantity of the cheese-milk and this was then added to the rest of the milk using a quantity of 0.4 g glucan/100 g cheese.

### 2.2. Cheese-Making Procedure

Three cheese-making trials took place in the pilot plant of the Dairy Research Department of the Institute of Technology of Agricultural Products, for the manufacture of a fresh, spreadable, and soft cheese. In each cheese-making trial, a quantity of 7 kg of ovine milk fortified with β-glucan was heated to 85–90 °C for 10 min. After cooling to 30 °C, the milk was inoculated with a commercial freeze-dried concentrated mesophilic culture (containing *Lactococcus lactis* subsp. *cremoris* and *Lactococcus lactis* subs. *lactis*). Then, powdered calf rennet (HALA, Hansen) at a quantity of 0.25 g for 100 kg milk was added. Coagulation stopped when the pH was 4.4 (this step lasted approximately 20 h). Then, the curd was placed in cloth bags and left for a 6 h drainage at 15 °C. The cheeses were removed from the cloth bags and were dry salted with 1.5% NaCl. Afterwards, cheeses were packed into plastic pots and transferred to cold storage (3–4 °C) for a period of 3weeks. Cheeses produced with β-glucan are designated as GC cheeses. The same manufacturing procedure was applied without fortification of the cheese-milk with β-glucan (control) and the resultant cheeses are referred to as CC cheeses.

The cheeses were analyzed for their physicochemical characteristics, sensory properties, proteolysis, lipolysis, color, viscosity, and antioxidant activity on different sampling dates (day-2, day-7, day-14, and day-21) and the reported results are, therefore, the averages of the 3 cheese-making trials.

### 2.3. Physicochemical Characteristics

The moisture content was determined by heating the cheese to a constant weight at 105 °C [18], the fat according to the Gerber–Van Gulik method [19], the salt using the modified Volhard method [20], and the ash following the method of IDF [21]. The cheese acidity was determined with the method described by the AOAC [22] and the pH of the cheeses was measured using a pH meter Micro pH 2002 (Crison, Barcelona, Spain). All materials used for the analyses in this study were of analytical grade.

The moisture in non-fat substance (MNFS) content was calculated by the equation:

MNFS% = Moisture% × 100/100 − Fat% (1)

The fat in dry matter (FDM) was calculated by the equation:

MNFS% = Fat% × 100/100 − Moisture% (2)

The salt-in-moisture (SM) was calculated by the equation:

SM% = Salt% × 100/Moisture% (3)

The protein content was calculated by the equation:

Protein% = Total Nitrogen% × 6.38 (4)

### 2.4. Sensory Properties

On different storage days, cheese samples were assessed organoleptically by five trained panel members who were permanent staff of the Dairy Research Department and familiar with this type of cheese. During the evaluation at ambient temperature (18 ± 2 °C), the samples were placed in white plastic plates coded with random 3-digit numbers. Water was provided for mouth washing. The panelists were asked to report any defects using the quality terms of the IDF [23] guide for the sensory evaluation of cheese and evaluated cheese samples on their appearance, texture, and flavor. All the examined attributes were scored on a 0–10 scale (0 = lowest quality, 10 = best quality). The scores for appearance, texture, and flavor were multiplied by a factor 1, 4, and 5, respectively, according to the importance given for each attribute. The total score was obtained by adding the scores of the individual attributes. An excellent cheese would receive a total score of 100.

### 2.5. Proteolysis

The total nitrogen (TN) content, water-soluble nitrogen (WSN), and nitrogen soluble in 12% trichloroacetic acid (TCA-N) and in 5%phosphotungstic acid (PTA-N) were determined using the Kjeldahl method [24]. The WSN and the TCA-N were determined in cheese extracts as described by Kuchroo and Fox [25] with the following modifications: Whatman No 42 filter paper was used for the filtration and the Sorval Omni-Mixer (Dupont Company, Newton, CT, USA) was used for sample homogenization. The nitrogen soluble in 5% phosphotungstic acid (PTA) was determined by the Kjeldahl method according to Stadhouders [26], with the exception that the extract was prepared as mentioned above.

### 2.6. Lipolysis 

Free fatty acids (FFAs) were extracted according to the modified method of De Jong and Badings [27]. Nonanoic acid (C9:0) was used as an internal standard. Gas chromatography was carried out with a Shimadzu model GC2010 (Shimadzu Scientific Instruments Inc., Columbia, MD, USA), equipped with a flame ionization detector (FID). The FFAs were separated using the column SGEBP21-FFAP (15 m × 0.53 mm × 0.5 µm i.d.). The concentration of the total FFAs was calculated by adding the concentrations of the individual FFAs.

### 2.7. Color and Viscosity Measurements

The color of the analyzed cheese samples was measured using a Hunter Lab DP-9000 (Hunter Associates Laboratory, Inc., Reston, VA, USA) colorimeter. Three color parameters were determined, i.e., L* (lightness), a* (green-red) value, and b* (blue-yellow) value, according to the CIELAB color space.

The viscosity measurements of the cheese samples were performed using a viscometer (Brookfield Engineering Laboratories Inc., Stoughton, MA, USA; model RVT) at 4 °C, using a spindle No 7, with a speed of 2.5 rpm.

### 2.8. Antioxidant Activity

The radical scavenging activity of 2,2-diphenyl-1-picrylhydrazyl (DPPH), the ferrous ion chelating activity, and the superoxide anion (SOSA) scavenging activity of the water-soluble cheese extracts were assayed using the methods of Gupta et al. [28] and Meira et al. [29]. The water-soluble extract was prepared as described above (section proteolysis).

### 2.9. Statistical Analysis

One-way analysis of variance was used for the comparison of the data of each parameter of the cheeses manufactured with or without fortification with β-glucans on the same storage day. Statistical analysis was performed using statistical package STATGRAPHICS Plus for Windows version 5.2, Manugistics Inc., Rockville, MD, USA. The results were considered statistically significant when *p* ≤ 0.05 [30].

## 3. Results and Discussion

### 3.1. Physicochemical Characteristics

The mean values of the physicochemical properties of GC and CC cheeses are presented in Table 1. Generally, these values are in accordance with those reported for similar types of cheeses, i.e., Galotyri [31,32], galotyri-type [33,34], Katiki Domokou [31], and Labneh [35]. However, some differences were observed between the mean values of the cheeses of the present work and those reported by other authors. Katsiari et al. [33] and Kondyli et al. [34] reported lower acidity values for galotyri-type cheeses while Zoidou et al. [32] found a lower ash content for Galotyri cheese when compared to the values of this study. Moreover, Danezis et al. [31], Anifantakis [36], and Lekkas et al. [37] measured lower pH values compared to the values reported in Table 1 for ovine soft spreadable cheese. The different compositions of the cheese-milk and of the conditions used during cheese production may explain the above differences.

In this study, the salt content of cheeses ranged from1.39–1.71% (Table 1), in accordance with the results for galotyri-type cheese [33,34] but lower than that determined by Anifantakis [36] for Galotyri cheese. Therefore, the cheeses of the present study meet the current consumers’ demand to consume less salt from nutrition.

In general, from Table 1, it can be seen that no significant differences were found regarding the composition of the GC and CC cheeses. Similarly, no differences were observed by Kondyli et al. [15] for white-brined cheese with or without the fortification with beta-glucan. However, cheese with β-glucan exhibited lower fat-in-dry matter levels than control cheese (Table 1) on the 7thand 14th day of storage. Moreover, GC cheeses showed higher moisture levels than CC cheeses probably due to the high water-binding ability of β-glucan [38,39]. Similar results were, generally, found by other authors [15,40,41].

### 3.2. Color and Viscosity Measurements

The color of a food product is an important parameter as it has a direct effect on its acceptability by consumers. Concerning the color parameters of the ovine soft spreadable cheeses manufactured with or without fortification with 0.4% beta-glucan, it can be seen (Table 2) that no differences (*p* > 0.05) were observed between the cheeses, indicating the same luminous yellow and green color, as denoted from the L*, b*, and a* parameters, respectively. Similarly, Kondyli et al. [15] generally did not observe significant differences in the color measurements of low-fat, white-brined cheese made with or without the fortification with 0.4% β-glucan sourced from mushroom. Moreover, Singh et al. [42] reported that β-glucan at 0.1–0.3% had no impact on yoghurt color but a further increase in its amount caused a decline in L* and an increment in the a* and b* parameters.

The rheological behavior of a food is crucial in order to maintain quality control of its ingredients and the final product and to develop food products that are acceptable to consumers [43]. The viscosity values of the fresh spreadable GC and CC cheeses of the present study are presented in Table 2. These values were higher than those observed for yogurt made with β-glucan [14], as expected since an amount of rennet was used for the curdling of the cheese-milk. Usually, rennet is added to the milk that is used for the manufacture of fresh cheeses as it improves the curd drainage and increases its firmness [44]. No significant differences were found between GC and CC cheeses regarding their viscosity. Similarly, Pappa et al. [14] found that yoghurt containing 0.3% β-glucan had the same viscosity as the control. Khorshidian et al. [9] reported that the incorporation of β-glucan at 1% and higher levels into the formulation of yoghurt enhanced the apparent viscosity compared to control samples. Additionally, Mejri et al. [45] observed that increasing the amount of β-glucan in yoghurt resulted in a significant increase in the viscosity only when the yoghurt contained 1.5% β-glucan.

### 3.3. Sensory Evaluation

The sensory properties represent a crucial factor that affects the consumer’s decision to purchase a cheese; therefore, it is important that added ingredients do not negatively affect the sensory properties. Table 3 presents the sensory evaluation of the fresh, soft, and spreadable GC and CC cheeses. The production method used in this study meant that both GC and CC cheeses received very high scores for all the organoleptic characteristics and were very much appreciated by the panelists, as shown in Table 3. No differences (*p* > 0.05) between the GC and CC cheeses were observed regarding the appearance and texture scores. However, the GC cheeses received significantly higher values for flavor than CC cheeses on the 14th day and 21st day.

Controversial results regarding the sensory evaluation of dairy products fortified with β-glucan exist. Pappa et al. [14] and Kondyli et al. [15] observed higher flavor values for yoghurt and the reduced-fat white-brined cheese with β-glucan in comparison to control samples. On the contrary, the use of β-glucan negatively influence the flavor of Kashar cheese [46], Cheddar cheese [47], and low-fat white-brined cheese [40]. Generally, the supplementation of dairy products with β-glucan higher than 1% has a negative effect on their sensory characteristics [9].

The panelists reported that all cheeses in this study had a soft and spreadable texture and a sourish, refreshing, mild flavor and acidic taste. Moreover, no defects regarding the appearance (such as whey separation), body-texture (such as weak body), or flavor (such as bitterness or rancid) were noted by the panelists.

### 3.4. Proteolysis

Proteolysis can be measured by determining different nitrogen soluble fractions, such as WSN, 12% TCA fraction, and 5% PTA fraction. It is known that WSN contains whey proteins, peptides with low molecular weight, and free amino acids [48]; 12% TCA fraction contains peptides with 2–20 residues and free amino acids [49]; and 5% PTA fraction contains peptides with molecular weight <600 Daltons and free amino acids [50]. Milk clotting enzymes and indigenous milk enzymes are responsible for the initial degradation of caseins that results in the formation of large- and medium-sized peptides. These peptides are subsequently hydrolyzed by enzymes from different sources (coagulant, starter, and nonstarter microorganisms) [51].

Table 4 presents the changes in the TN% and in the soluble fractions, i.e., WSN, TCA, and PTA of GC and CC fresh spreadable cheeses. Higher WSN values were determined in the cheeses of the present study than those reported for 15-day galotyri-type cheese [34] and Quarg, an acid-coagulated fresh cheese [52]. It is known that a significant part of WSN forms due to the action of rennet [53]; therefore, the above differences could be attributed to differences in the activity of the residual rennet.

In this study, the WSN%TN levels were 5.31–6.09 on the 2nd day and increased to 11.68–12.20 on the 21st day (Table 4), in accordance with the results of other authors for these types of cheeses [34,52]. The levels of TCA%TN, regardless of the fortification with β-glucan, were 5.27–5.76 on day-2 and 5.29–5.75 on day-21. The levels of PTA%TN were 2.98–2.59 on the 2nd day and 3.02–2.98 on the 21st day (Table 4). A small increase in PTA-N was observed possibly because of the low pH and storage temperature (3–4 °C) of cheeses, which did not favor the activity of enzymes in the starter microorganisms.

From Table 4, it can be seen that generally, no differences (*p* > 0.05) regarding the proteolysis levels were observed between GC and CC cheeses. Similarly, no differences were determined by Kondyli et al. [15] in white-brined cheeses fortified with β-glucan. On the contrary, generally, Sahan et al. [46] observed higher proteolysis levels in Kashar cheeses and Volikakis et al. [40] in white-brined cheeses fortified with β-glucan than control cheeses. These differences might be attributed to the different conditions used during cheese production, ripening, and storage.

### 3.5. Lipolysis

Free fatty acids (FFAs) are important components of the flavor of many cheese types. The type of cheese-milk, heat treatment, cultures used, ripening and storage conditions, and lipases of the rennet are some factors that affect the FFA concentration of a cheese [53].

The acetic acid and FFA levels in GC and CC cheeses are shown in Table 5. Acetic acid contributes to the flavor of cheeses that are produced by acid or acid/rennet coagulation, although it is not a product of lipolysis but of other biochemical pathways [54,55].

The free fatty acids C2 (acetic), C12 (lauric), and C16 (palmitic) were found in abundance at all ages for both cheeses (Table 5). These free fatty acids were found in high levels in similar cheese varieties [33,56].

The levels of FFAs in GC and CC cheeses did not differ significantly at all sampling ages, except on the 7th day, the CC cheeses showed lower levels (*p* < 0.05) than GC cheeses. Moreover, Kondyli et al. [15] found that, generally, low-fat white-brined cheeses produced with or without fortification with β-glucan during ripening and storage did not significantly differ in the total FFA content, except on day-180. However, Sahan et al. [46] observed higher lipolysis levels of Kashar cheeses with beta-glucan than cheeses without it.

### 3.6. Antioxidant Activity

The hydrophilic fraction of milk or cheese, which contains peptides, water-soluble vitamins, etc., and the hydrophobic fraction, which consists of compounds, such as conjugated linoleic acid and vitamins A and D3, are responsible for its antioxidant activity [57].

The results of the different methods used to evaluate the antioxidant activity of GC and CC cheeses are presented in Table 6. Τhe DPPH assay remained almost constant during storage (54.85–54.90%RSA onday-2 and 57.40–57.03%RSA onday-21 in CC and GC cheeses, respectively). The reference antioxidant Trolox at the 0.25 mg/mL concentration showed 93.5% DPPH scavenging activity. Similarly, Moschopoulou et al. [58] determined a high antioxidant capacity (58–70%) in yoghurts during 21 days of storage; however, low DPPH radical scavenging activity (26%) was observed in cottage fresh cheese during 28 days of storage by Kariyawasam et al. [59]. When the Fe^+2^ chelating activity method was performed, the results showed that both CC and GC cheeses exhibited high chelating activity >60% (Table 6) on all sampling dates. The reference standard EDTA at a concentration of 0.1 mg/mL could chelate 99.9% of the available iron. From Table 6, it can be seen that the superoxide scavenging activity was high in both CC and GC cheeses, ranging from 69.42–73.01% onday-2 to 81.25–87.80% onday-21, respectively, and the standard Trolox reference antioxidant at a concentration of 0.25 mg/mL showed an 86.33% superoxide scavenging activity.

It is known that the significant antioxidant properties of mushrooms can be attributed to their bioactive compounds (i.e., polyphenols, carotenoids, etc.) [60]. From Table 6, it can be seen that there were no significant differences between the GC and CC cheeses during storage, regardless of the method used to determine the antioxidant capacity. Further analysis might be necessary to determine the antioxidant activity of the beta-glucan paste.

## 4. Conclusions

As the consumer’s demand for healthy nutrition is increasing, the development of new food products with properties that strengthen human health is necessary. The data obtained from this study showed that β-glucan extracted from *Pleurotus ostreatus* mushrooms could be successfully incorporated into a fresh, soft, and spreadable cheese made of ovine milk in the form of paste at a concentration 0.4 g glucan for 100 g of cheese. The results showed that at this concentration, generally, β-glucan did not significantly affect the composition and biochemical properties of the resultant cheeses, which were very much appreciated by the panelists during the sensory evaluation. However, cheeses fortified with β-glucan showed a higher moisture content than control cheeses on the1st and 21st days of storage while the levels of proteolysis and the sensory properties of the cheeses were unaffected. The successful manufacture of a functional spreadable cheese fortified with β-glucan is of great interest to food manufacturers. Future research will focus on the composition of the beta glucan paste, its biochemical characteristics, its effect on the microbiological properties of cheeses, and the health benefits of cheeses fortified with β-glucan in clinical studies.

## Figures and Tables

**Table 1:** Composition and physicochemical properties of GC and CC fresh spreadable cheeses made from ovine milk.

Storage Time (Days)	Cheese	pH	Moisture (%)	Moisture in Non-Fat Substance (%)	Fat (%)	Fat in Dry Matter (%)	Salt (%)	Salt-in-Moisture (%)	Acidity (% Lactic Acid)	Ash (%)	Proteins (%)
2	CC	4.34 ± 0.02 ^a^	72.60 ± 0.36 ^a^	82.50 ± 0.50 ^a^	12.00 ± 0.58 ^a^	43.76 ± 0.93 ^a^	1.41 ± 0.12 ^a^	1.94 ± 0.14 ^a^	1.46 ± 0.04 ^a^	2.26 ± 0.04 ^a^	10.45 ± 0.49 ^a^
	GC	4.37 ± 0.04 ^a^	74.49 ± 0.66 ^b^	83.32 ± 0.19 ^a^	10.60 ± 0.60 ^a^	41.49 ± 1.23 ^a^	1.44 ± 0.15 ^a^	1.94 ± 0.21 ^a^	1.37 ± 0.03 ^a^	2.33 ± 0.04 ^a^	9.12 ± 0.41 ^a^
7	CC	4.39 ± 0.03 ^a^	72.22 ± 0.84 ^a^	81.79 ± 0.44 ^a^	11.70 ± 0.56 ^a^	42.07 ± 0.71 ^a^	1.50 ± 0.13 ^a^	2.08 ± 0.18 ^a^	1.46 ± 0.06 ^a^	2.30 ± 0.04 ^a^	10.14 ± 0.66 ^a^
	GC	4.38 ± 0.02 ^a^	74.40 ± 0.55 ^a^	82.70 ± 0.40 ^a^	10.03 ± 0.23 ^a^	39.19 ± 0.08 ^b^	1.48 ± 0.07 ^a^	1.99 ± 0.08 ^a^	1.46 ± 0.06 ^a^	2.30 ± 0.01 ^a^	8.60 ± 0.29 ^a^
14	CC	4.33 ± 0.06 ^a^	72.14 ± 1.07 ^a^	81.97 ± 0.76 ^a^	12.00 ± 0.50 ^a^	43.06 ± 0.24 ^a^	1.45 ± 0.00 ^a^	2.01 ± 0.03 ^a^	1.46 ± 0.06 ^a^	2.32 ± 0.04 ^a^	9.60 ± 0.28 ^a^
	GC	4.32 ± 0.05 ^a^	74.34 ± 0.54 ^a^	82.75 ± 0.37 ^a^	10.17 ± 0.44 ^a^	39.60 ± 1.16 ^b^	1.39 ± 0.06 ^a^	1.87 ± 0.10 ^a^	1.38 ± 0.02 ^a^	2.25 ± 0.01 ^a^	8.81 ± 0.23 ^a^
21	CC	4.45 ± 0.04 ^a^	72.16 ± 0.78 ^a^	81.54 ± 0.41 ^a^	11.50 ± 0.58 ^a^	41.27 ± 1.05 ^a^	1.55 ± 0.06 ^a^	2.14 ± 0.08 ^a^	1.44 ± 0.06 ^a^	2.32 ± 0.03 ^a^	9.54 ± 0.37 ^a^
	GC	4.41 ± 0.03 ^a^	75.26 ± 0.14 ^b^	85.14 ± 1.71 ^a^	10.30 ± 0.50 ^a^	43.97 ± 4.36 ^a^	1.71 ± 0.18 ^a^	2.24 ± 0.26 ^a^	1.41 ± 0.06 ^a^	2.22 ± 0.03 ^a^	8.50 ± 0.17 ^a^

GC: cheeses fortified with β-glucan; CC: control cheeses (without fortification withβ-glucan). Means of three replicates ± standard error. For each parameter in the same column and time, different letters indicate statistically significant differences between the cheeses (*p* < 0.05).

**Table 2:** Measurements of the color and viscosity of GC and CC fresh spreadable cheeses made from ovine milk.

Storage Time (Days)	Cheese	L*	a*	b*	Viscosity, cP (mPa*s)
2	CC	95.00 ± 0.45 ^a^	−24.21 ± 0.97 ^a^	8.40 ± 0.21 ^a^	320,000 ± 32,000 ^a^
	GC	92.95 ± 1.05 ^a^	−22.76 ± 1.03 ^a^	8.19 ± 0.53 ^a^	282,667 ± 14,111 ^a^
7	CC	95.31 ± 0.32 ^a^	−24.61 ± 0.36 ^a^	9.30 ± 0.24 ^a^	474,667 ± 28,221 ^a^
	GC	93.69 ± 0.53 ^a^	−23.95 ± 0.90 ^a^	8.59 ± 0.37 ^a^	412,333 ± 57,713 ^a^
14	CC	95.02 ± 0.40 ^a^	−24.93 ± 0.76 ^a^	10.42 ± 0.23 ^a^	421,333 ± 5333 ^a^
	GC	94.51 ± 0.26 ^a^	−24.16 ± 0.74 ^a^	9.89 ± 0.70 ^a^	400,000 ± 18,475 ^a^
21	CC	93.22 ± 1.71 ^a^	−22.92 ± 0.72 ^a^	10.23 ± 0.38 ^a^	405,333 ± 19,230 ^a^
	GC	92.99 ± 0.40 ^a^	−24.49 ± 0.59 ^a^	10.92 ± 0.83 ^a^	320,000 ± 64,000 ^a^

GC: cheeses fortified with β-glucan; CC: control cheeses (without fortification with β-glucan). Means of three replicates ± standard error. For each parameter in the same column and time, the same letters indicate no statistically significant differences between the cheeses (*p* > 0.05).

**Table 3:** Organoleptic characteristics of GC and CC fresh spreadable cheeses made from ovine milk.

Storage Time (Days)	Cheese	Appearance (10)	Texture (40)	Flavour (50)	Total (100)
2	CC	9.14 ± 0.14 ^a^	36.35 ± 0.35 ^a^	44.49 ± 0.32 ^a^	90.30 ± 0.80 ^a^
	GC	8.80 ± 0.10 ^a^	36.80 ± 0.07 ^a^	45.20 ± 0.47 ^a^	90.80 ± 0.61 ^a^
7	CC	8.97 ± 0.07 ^a^	35.72 ± 0.62 ^a^	44.03 ± 0.97 ^a^	89.63 ± 0.63 ^a^
	GC	8.90 ± 0.10 ^a^	36.33 ± 0.20 ^a^	45.13 ± 0.35 ^a^	90.40 ± 0.21 a
14	CC	8.77 ± 0.09 ^a^	35.95 ± 0.55 ^a^	40.97 ± 1.74 ^a^	87.55 ± 0.50 ^a^
	GC	9.03 ± 0.09 ^a^	36.60 ± 0.17 ^a^	45.37 ± 0.37 ^b^	90.97 ± 0.41 ^a^
21	CC	8.93 ± 0.15 ^a^	34.80 ± 0.40 ^a^	41.83 ± 0.88 ^a^	84.60 ± 1.00 ^a^
	GC	9.13 ± 0.13 ^a^	36.93 ± 0.48 ^a^	45.17 ± 1.01 ^b^	91.23 ± 1.59 ^a^

GC: cheeses fortified with β-glucan; CC: control cheeses (without fortification with β-glucan). The maximum scores of each value are reported in brackets. Means of three replicates ± standard error. For each parameter in the same column and time, the same letters indicate no statistically significant differences between the cheeses (*p* > 0.05).

**Table 4:** Proteolysis of GC and CC fresh spreadable cheeses made from ovine milk.

Storage Time (Days)	Cheese	TN (%)	WSN (%TN)	TCA (%TN)	PTA (%TN)
2	CC	1.64 ± 0.08 ^a^	5.31 ± 0.27 ^a^	5.27 ± 0.30 ^a^	2.98 ± 0.42 ^a^
	GC	1.43 ± 0.06 ^a^	6.09 ± 0.39 ^a^	5.76 ± 0.11 ^a^	2.59 ± 0.05 ^a^
7	CC	1.59 ± 0.10 ^a^	5.53 ± 0.12 ^a^	4.98 ± 0.07 ^a^	2.48 ± 0.12 ^a^
	GC	1.35 ± 0.05 ^a^	6.96 ± 0.48 ^b^	5.24 ± 0.63 ^a^	2.61 ± 0.35 ^a^
14	CC	1.50 ± 0.04 ^a^	10.12 ± 1.12 ^a^	4.99 ± 0.42 ^a^	2.90 ± 0.34 ^a^
	GC	1.38 ± 0.04 ^a^	10.42 ± 1.49 ^a^	4.91 ± 0.29 ^a^	2.47 ± 0.15 ^a^
21	CC	1.49 ± 0.06 ^a^	11.68 ± 0.24 ^a^	5.29 ± 0.26 ^a^	3.02 ± 0.36 ^a^
	GC	1.34 ± 0.03 ^a^	12.20 ± 0.31 ^a^	5.75 ± 0.21 ^a^	2.98 ± 0.17 ^a^

GC: cheeses fortified with β-glucan; CC: control cheeses (without fortification with β-glucan). Means of three replicates ± standard error. For each parameter in the same column and time, different letters indicate statistically significant differences between the cheeses (*p* < 0.05).

**Table 5:** Lipolysis of GC and CC fresh spreadable cheeses made from ovine milk.

FFA(μg/g)	2nd Day of Storage		7th Day of Storage		14th Day of Storage		21st Day of Storage	
	CC	GC	CC	GC	CC	GC	CC	GC
Acetic acid-C2	94.53 ± 14.03 ^a^	161.52 ± 5.63 ^b^	95.44 ± 5.35 ^a^	159.07 ± 25.76 ^b^	116.42 ± 0.51 ^a^	158.01 ± 21.94 ^b^	129.20 ± 9.01 ^a^	167.21 ± 14.95 ^b^
Butyric acid-C4	16.61 ± 5.48 a	11.8 ± 1.67 ^a^	19.3 ± 1.52 ^a^	12.9 ± 2.00 ^b^	18.5 ± 1.70 ^a^	15.37 ± 3.09 ^a^	19.5 ± 2.52 ^a^	18.4 ± 1.03 ^a^
Isobutyric acid-C4 ISO	2.73 ± 0.12 ^a^	1.84 ± 0.11 ^b^	2.58 ± 0.33 ^a^	3.16 ± 0.51 ^a^	4.20 ± 1.69 ^a^	2.58 ± 0.37 ^a^	6.13 ± 2.41 ^a^	3.59 ± 0.66 ^a^
Isovaleric acid-C5 ISO	3.09 ± 0.15 ^a^	2.70 ± 0.16 ^a^	3.59 ± 0.67 ^a^	3.54 ± 0.25 ^a^	5.31 ± 2.00 ^a^	3.60 ± 0.52 ^a^	5.59 ± 1.73 ^a^	6.80 ± 0.59 ^b^
Caproic acid-C6	5.81 ± 0.54 ^a^	5.93 ± 0.53 ^a^	6.37 ± 0.50 ^a^	6.10 ± 0.54 ^a^	6.08 ± 0.34 ^a^	6.80 ± 0.59 ^a^	7.07 ± 1.45 ^a^	5.65 ± 0.72 ^a^
Caprylic acid-C8	2.02 ± 0.65 ^a^	1.33 ± 0.28 ^a^	1.38 ± 0.09 ^a^	1.02 ± 0.02 ^b^	1.32 ± 0.30 ^a^	1.38 ± 0.28 ^a^	2.92 ± 1.18 ^a^	1.72 ± 0.03 ^a^
Capric acid-C10	4.03 ± 1.22 ^a^	3.78 ± 0.86 ^a^	3.85 ± 0.3 ^a^	4.35 ± 0.8 ^a^	5.42 ± 1.48 ^a^	4.85 ± 0.55 ^a^	4.23 ± 1.38 ^a^	7.28 ± 1.42 ^a^
Lauric acid-C12	47.85 ± 9.59 ^a^	42.45 ± 3.65 ^a^	53.55 ± 2.37 ^a^	48.33 ± 4.23 ^a^	53.75 ± 6.35 ^a^	44.88 ± 1.89 ^a^	51.22 ± 3.13 ^a^	48.45 ± 5.37 ^a^
Myristic acid-C14	11.02 ± 1.55 ^a^	10.62 ± 0.84 ^a^	10.33 ± 2.03 ^a^	11.97 ± 0.95 ^a^	12.83 ± 1.47 ^a^	13.65 ± 2.33 ^a^	12.07 ± 0.66 ^a^	18.72 ± 1.67 ^b^
Myristoleic Acid-C14:1	1.35 ± 0.65 ^a^	0.93 ± 0.13 ^a^	1.28 ± 0.13 ^a^	1.32 ± 0.25 ^a^	1.15 ± 0.22 ^a^	1.43 ± 0.15 ^a^	1.22 ± 0.06 ^a^	1.07 ± 0.19 ^a^
Palmitic acid-C16	50.52 ± 12.04 ^a^	42.77 ± 5.97 ^a^	56.1 ± 6.21 ^a^	49.82 ± 10.29 ^a^	53.88 ± 9.38 ^a^	50.1 ± 2.19 ^a^	57.52 ± 1.20 ^a^	61.3 ± 5.07 ^a^
Palmitoleic acid-C16:1	7.65 ± 1.88 ^a^	3.75 ± 0.40 ^a^	8.52 ± 0.61 ^a^	4.67 ± 0.89 ^a^	8.43 ± 0.23 ^a^	6.73 ± 0.83 ^a^	8.17 ± 1.55 ^a^	6.85 ± 1.98 ^a^
Stearic acid-C18	23.83 ± 3.08 ^a^	17.48 ± 1.67 ^a^	28.67 ± 2.11 ^a^	18.68 ± 0.78 ^a^	30.9 ± 3.0 ^a^	25.53 ± 6.18 ^a^	33.23 ± 1.28 ^a^	27.7 ± 3.16 ^a^
Oleic acid-C18:1	16.73 ± 0.73 ^a^	12.43 ± 2.33 ^a^	14.78 ± 3.23 ^a^	11.82 ± 0.59 ^b^	19.47 ± 0.98 ^a^	12.8 ± 1.67 ^a^	21.68 ± 0.83 ^a^	19.18 ± 5.78 ^a^
Linoleic acid-C18:2	N.D.	N.D.	N.D.	N.D.	3.32 ± 1.18 ^a^	1.62 ± 0.43 ^a^	2.8 ± 1.43 ^a^	1.75 ± 0.45 ^a^
TFFA	287.77 ± 13.89 ^a^	319.33 ± 11.72 ^a^	305.74 ± 10.52 ^a^	386.75 ± 23.42 ^b^	340.98 ± 20.55 ^a^	349.52 ± 20.87 ^a^	362.55 ± 19.9 ^a^	395.67 ± 20.57 ^a^

GC: cheeses fortified with β-glucan; CC: control cheeses (without fortification with β-glucan). N.D.: Not Detected. Means of three replicates ± standard error. For each parameter in the same row and time, different letters indicate statistically significant differences between the cheeses (*p* < 0.05).

**Table 6:** Antioxidant capacity of GC and CC fresh spreadable cheeses made from ovine milk.

Storage Time (Days)	Cheese	DPPH% RSA	%CA (Fe^+2^)	SOSA%
2	CC	54.85 ± 7.22 ^a^	64.63 ± 2.74 ^a^	69.42 ± 4.24 ^a^
	GC	54.90 ± 4.47 ^a^	65.06 ± 2.06 ^a^	73.01 ± 5.12 ^a^
7	CC	55.44 ± 1.39 ^a^	63.13 ± 2.52 ^a^	77.68 ± 6.70 ^a^
	GC	51.47 ± 2.51 ^a^	61.37 ± 1.77 ^a^	70.98 ± 1.34 ^a^
14	CC	54.07 ± 3.24 ^a^	71.36 ± 4.38 ^a^	77.23 ± 4.77 ^a^
	GC	52.65 ± 3.78 ^a^	68.38 ± 2.52 ^a^	79.76 ± 1.95 ^a^
21	CC	57.40 ± 3.59 ^a^	70.47 ± 3.65 ^a^	81.25 ± 3.81 ^a^
	GC	57.03 ± 6.71 ^a^	71.30 ± 0.20 ^a^	87.80 ± 0.83 ^a^

GC: cheeses fortified with β-glucan; CC: control cheeses (without fortification with β-glucan). Means of three replicates ± standard error. For each parameter in the same column and time, the same letters indicate no statistically significant differences between the cheeses (*p* > 0.05).
